# Initial Low-Dose Hydroxyurea and Anagrelide Combination in Essential Thrombocythemia: Comparable Response with Lower Toxicity

**DOI:** 10.3390/jcm13102901

**Published:** 2024-05-14

**Authors:** Young Hoon Park, Yeung-Chul Mun, Sewon Lee, Yongchel Ahn

**Affiliations:** 1 Division of Hematology-Oncology, Department of Internal Medicine, Ewha Womans University Medical Center Mokdong Hospital, Seoul 07985, Republic of Korea; carrox2yh@gmail.com (Y.H.P.); yeungchul@ewha.ac.kr (Y.-C.M.); swclara@gmail.com (S.L.); 2 Department of Hematology and Oncology, Gangneung Asan Hospital, University of Ulsan College of Medicine, Gangneung 25440, Republic of Korea

**Keywords:** essential thrombocythemia, hydroxyurea, anagrelide

## Abstract

**Background and Objectives:** Essential thrombocythemia (ET) is a myeloproliferative neoplasm that overproduces platelets and is associated with life-threatening thrombosis. Medical cytoreduction either with hydroxyurea (HU) or anagrelide (AG) is widely used, but drug intolerance or resistance are major concerns. Low-dose combination of HU and AG as an alternative strategy has been explored in various studies. It showed comparable response with acceptable toxicity in second-line settings for patients who experienced side effects from prior monotherapy. In this study, we evaluated the efficacy and safety of upfront combination for ET patients. **Materials and Methods:** From January 2018 to June 2022, a total of 241 ET patients with intermediate to high risk were enrolled. We identified 21 patients with initial drug combinations and compared treatment outcomes and adverse events (AEs) between combination and monotherapy groups. **Results:** The median age was 62 years old (range, 26–87) and median platelet count was 912 × 10^9^/L (range, 520–1720). Overall treatment response did not exhibit significant differences between the groups, although there was a trend towards a lower response rate in patients treated with AG alone at 3 months post-treatment (AG + HU, 85.7% vs. AG alone, 75.4%, *p* = 0.068). AEs of any grade occurred in 52.3% of the combination group, 44.3% of the HU monotherapy group, and 43.4% of the AG single group, respectively. Of note was that the HU plus AG combination group suffered a lower incidence of grade 3–4 AEs compared to the other two groups, with statistical significance (*p* = 0.008 for HU monotherapy vs. combination therapy and *p* < 0.01 for AG monotherapy vs. combination therapy). **Conclusions:** Our findings demonstrated that the upfront low-dose combination approach showed feasible clinical outcomes with significantly lower severe AEs compared to conventional monotherapy. These results may offer valuable insights to clinicians for future prospective investigations.

## 1. Introduction

Essential thrombocythemia (ET) is a chronic Philadelphia-negative myeloproliferative neoplasm and is associated with a high risk of thrombotic complications and transformation to myelofibrosis (MF) or acute myeloid leukemia (AML) [1,2]. The primary therapeutic goal is to prevent thrombotic or bleeding events without increasing the risk of disease transformation. This is achieved through cytoreductive interventions, guided by thrombosis risk assessment [2,3]. Traditionally, risk stratification in ET included old age (>60 years), thromboembolic history, and extreme thrombocytosis (platelet count > 1500 × 10^9^/L), and those who have any of these risk factors are categorized into high-risk patients. Based on evidence from three randomized studies [4,5,6], the European Leukemia Network (ELN) recommended hydroxyurea (HU) along with antiplatelet agents as a first-line treatment option for high-risk patients [7]. Anagrelide (AG), a selective platelet-reducing agent, is also recommended as a second-line treatment option for patients who are refractory or intolerant to HU, particularly when cytoreduction therapy is deemed necessary [6,7].

Nevertheless, approximately 20% of ET patients experience either intolerance or resistance to high-dose HU due to treatment-related toxicities, such as myelosuppression, fever, mucocutaneous manifestations, and leg ulcer [8,9]. And high-dose AG is also associated with poor tolerability, with up to 90% of patients experiencing variable adverse effects (AEs) including headache, diarrhea, tachycardia, and palpitation [6,10,11]. These significant AEs inevitably lead to drug interruptions, dose reduction, and subsequent insufficient cytoreduction, which is associated with an increased risk of suboptimal response, disease transformation, and even poor survival [10,12].

To address the above problems, some studies investigated the combination of HU and AG at a lower dose [11,12,13,14,15]. Given the different mechanisms of action of the two drugs, it was postulated that this approach may possibly yield a synergistic effect. These studies reported that two drug combinations effectively managed platelet count and improved tolerance through reducing daily doses of the two drugs. This suggested that low-dose HU plus AG may offer a viable option for ET patients who showed insufficient response to initial therapy. However, it is noteworthy that these studies primarily focused on patients who exhibited resistance or intolerance to monotherapy, often involving the therapeutic scheme to add AG to an initial HU regimen. 

To date, there have been few studies that assessed the efficacy and safety of frontline HU and AG combination at a low dose. Considering efficacy and tolerability of combination therapy in a second-line setting, we postulated that efficacy and safety could also be feasible in upfront combination since there are currently no established guidelines for combination therapy in ET.

## 2. Materials and Methods

### 2.1. Patient Selection

**Figure 1:**
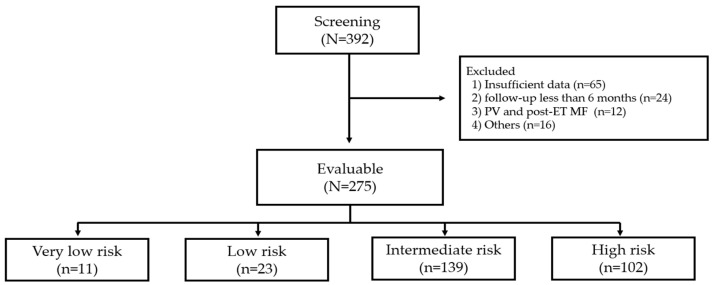
Flow diagram of the study. PV, polycythemia vera; ET, essential thrombocythemia; MF, myelofibrosis.

From January 2018 to June 2022, a total of 392 adult patients aged ≥ 18 years who were diagnosed with ET according to the World Health Organization (WHO) 2016 classification were screened (Figure 1) [1]. Patients were excluded if they had prefibrotic/early-stage primary myelofibrosis (MF), polycythemia vera, or post-ET MF, or had previously undergone therapy for ET other than HU or AG. According to the International Prognostic Score of thrombosis in World Health Organization-essential thrombocythemia (IPSET-thrombosis) model [3,16], risk stratification included four categories: very low risk (age ≤ 60 years, no previous thrombosis history, and JAK2 wild-type), low risk (age ≤ 60 years, no previous thrombosis history, and JAK2 mutated), intermediate risk (age > 60 years, no previous thrombosis history, and JAK2 wild-type), and high risk (age > 60 with mutated JAK2 or documented thrombosis history). Of the 275 evaluable patients, a total of 241 intermediate or high-risk patients (intermediate-risk, *n* = 139; high-risk, *n* = 102) were enrolled in this study. A retrospective review of patient’s medical records yielded the following clinical data: age at diagnosis, laboratory results including liver and renal function test, presence of JAK2 V617F mutation, history of prior thrombotic events, presence or absence of cardiovascular risk factors (hypertension, diabetes mellitus, and dyslipidemia), microvascular symptoms, daily dosage and duration of HU or AG treatment, treatment response, thrombotic or bleeding events during treatment, transformation to myelofibrosis or acute leukemia, the incidence of non-hematologic malignancies, and AEs. Allele-specific polymerase chain reaction (AS-PCR) was employed for the detection of the JAK2 V61F mutation in patients with ET, utilizing genomic DNA extracted from peripheral blood or bone marrow samples. The AS-PCR was carried out by using Forward wild-type primer (FC) 5′-ATC TAT AGT CAT GCT GAA AGT AGG AGA AAG-3′, Forward mutant primer (FM) 5′-AGC ATT TGG TTT TAA ATT ATG GAG TAT ATT-3′, and Reverse primer (FR) 5′-CTG AAT AGT CCT ACA GTG TTT TCA GTT TCA-3′ to cover the mutation point Val617Phe.

### 2.2. Treatment Scheme

Patients undergoing combination therapy received HU and AG at an initial dosage of 500 mg/day and 0.5 or 1 mg/day, respectively. The dosage of each drug was then titrated incrementally until the effective dose was determined. In the case where a patient experienced intolerance to either HU or AG, a dose reduction or switch to HU or AG monotherapy was permitted at the discretion of the physician. On the other hand, monotherapy patients typically started HU or AG at a dosage of 1000 mg/day and 1 mg/day, respectively. All the patients took anti-platelet medication, such as low-dose aspirin, unless contraindicated. 

### 2.3. Response Evaluation

Based on the ELN guideline, the criteria for response were defined as follows. (1) Complete response (CR) was defined as a platelet count of ≤400 × 10^9^/L, no disease-related symptoms, normal spleen size, and white blood cell count of ≤10 × 10^9^/L. (2) In patients who did not fulfill the criteria for complete response, platelet count of ≤600 × 10^9^/L or decrease of >50% from baseline was defined as partial response (PR). (3) No response (NR) was defined as any response that did not satisfy CR or PR [3,7]. Complete blood count monitoring was performed at least every 3 months and response assessment was performed at 3, 6, and 12 months after initiation of treatment. Adverse events were evaluated using the National Cancer Institute Common Toxicity Criteria (CTCAE) version 4.0 and recorded throughout the course of treatment. Resistance and intolerance to HU were classified in accordance with the ELN criteria for ET [8].

### 2.4. Statistical Analysis

Patient demographics and treatment subgroups were succinctly described using frequency tabulations for categorical variables and summary statistics for continuous variables. Continuous variables were listed by their medians with range, whereas categorical variables were reported as counts and percentages. Baseline characteristics and efficacy outcomes among the three treatment groups were compared employing the Pearson’s Chi-square test or Fisher’s exact test. Multivariate logistic regression analysis identified baseline characteristics associated with CR, providing odds ratio (OR) and 95% confidence intervals (95% CI) for the variables. All statistical tests were two-tailed, and significance was established at the level of *p* < 0.05. The statistical analysis for this study was performed using version 28 of the Statistical Package for the Social Sciences (SPSS Inc., Chicago, IL, USA).

## 3. Results

### 3.1. Patient Characteristics

The clinical characteristics of patients receiving frontline combination therapy are outlined in Table 1. The median duration from diagnosis to start of combination therapy was 5 days (range, 2–34 days). Fourteen patients (66.7%) were found to have JAK2 V617F gene mutation identified by AS-PCR methodology, while six (28.6%) reported microvascular symptoms such as lightheadedness. Hepatosplenomegaly was evaluated through physical examination or ultrasound/computer tomography imaging and was present in six patients (28.6%). Prior to enrollment, four patients (19.0%) had experienced thrombotic events (one transient ischemic attack, one cerebral infarction, and two deep vein thrombosis) and one patient had experienced intracranial hemorrhage. 

We conducted a comparison of the baseline characteristics among patients receiving HU and AG combination therapy versus those receiving HU or AG monotherapy (Table 1). There was a tendency for patients in the AG monotherapy group to be younger (*p* = 0.068) and for patients in the combination group to be at a high risk compared to the other groups (*p* = 0.081). However, we could observe no statistically significant differences in variables among the three groups. The incidence of prior thrombotic events was 20.5% for those on HU monotherapy and 17.4% for those on AG monotherapy. In this group, cerebral infarction and deep vein thrombosis were the most commonly reported thrombotic events in arterial and venous sites, respectively, mirroring the patterns observed in the combination therapy group.

### 3.2. Efficacy

**Figure 2:**
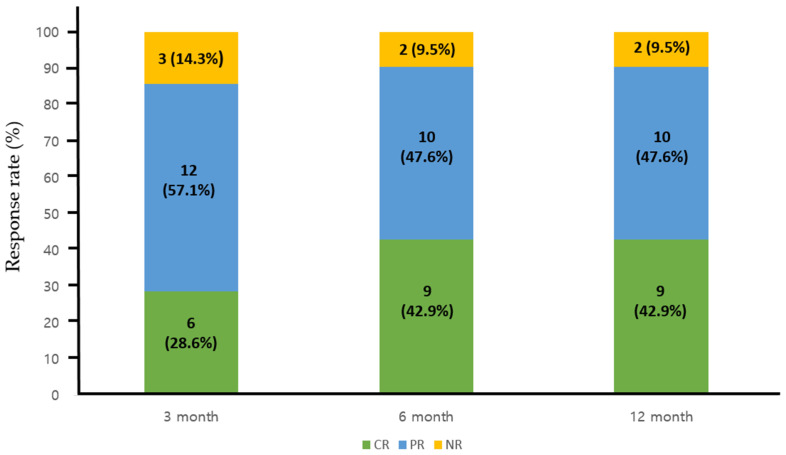
Response rate to the combination therapy at 3, 6, and 12 months post-treatment. CR, complete response; PR, partial response; NR, no response.

**Figure 3:**
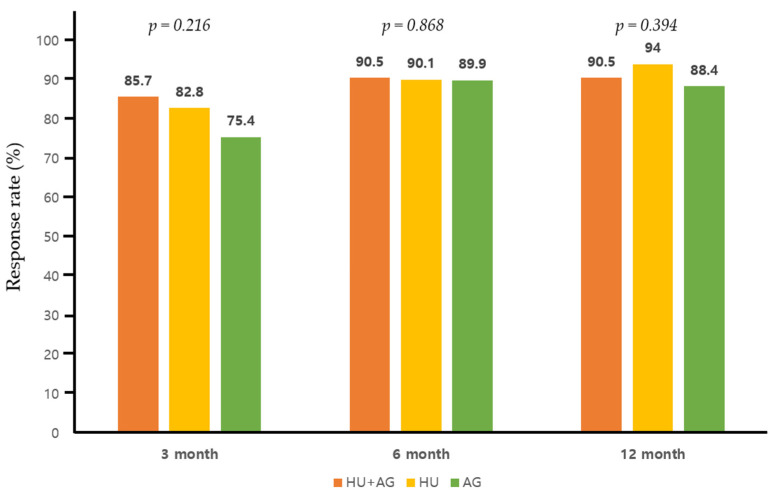
Overall response (complete and partial response) rate to each therapy at 3, 6, and 12 months post-treatment.

The treatment outcomes for each regimen are detailed in Table 2. In the combination group, the mean daily dose of HU and AG was 1142.5 mg/day (range, 500–2000) and 1.45 mg/day (range, 1.0–2.5), respectively, which were significantly lower than those administered in each monotherapy arm (*p* = 0.01 for HU and *p* < 0.01 for AG). Patients receiving AG monotherapy had a significantly shorter treatment duration compared to the other two groups (*p* = 0.022 for HU monotherapy). In terms of best overall treatment response at any time point in the combination group, we observed twelve CR patients (57.1%), eight PR patients (38.1%), and NR in one (4.8%). Specifically, for patients receiving combination therapy, the CR rate was 28.6%, 42.9%, and 42.9% at 3, 6, and 12 months after treatment, indicating a progressive improvement in objective response over the course of treatment (Figure 2). Overall response rate during the treatment period did not exhibit significant differences between the groups, although there was a trend towards a lower response rate in patients treated with AG alone at 3 months post-treatment (AG + HU, 18/21, 85.7% vs. AG alone, 57/69, 75.4%, *p* = 0.068, Figure 3). Within the combination group, only one patient experienced an arterial thrombotic event (cerebral infarction) after treatment. Thrombotic events occurred in nine patients (three arterial and six venous thrombosis) receiving HU monotherapy and in three patients (two arterial and one venous thromboses) receiving AG monotherapy, with no significant difference observed between the groups. During the respective courses of therapy, one patient experienced subdural hemorrhage in the HU monotherapy group, and two patients experienced bleeding events (pulmonary hemorrhage and small bowel bleeding) in the AG monotherapy group, while no bleeding events were reported in the combination therapy group (no statistical significance).

The observed rate of transformation, including MF, AML, and myelodysplastic syndrome, as well as the time from treatment initiation to transformation, were comparable to the other two groups. Additionally, during treatment, non-hematologic malignancies were observed in one patient in combination therapy (lung adenocarcinoma) and one patient in HU monotherapy (malignant melanoma).

In a multivariate logistic regression model, age > 70 years old (OR [95% CI]: 4.88 [1.684–20.418], *p* < 0.01) and the presence of JAK2 V617F mutation (OR [95% CI]: 3.24 [1.587–14.369], *p* < 0.01) were identified as significant predictors of CR evaluated at 1 year of treatment.

### 3.3. Safety and Tolerability

With a median follow-up of 3.1 years (range, 0.7–4.9 years), summarized toxicities in each patient group are presented in Table 3. Treatment-related adverse events of any grade occurred in 52.3% of patients receiving HU plus AG combination, 44.3% of those in HU monotherapy, and 43.4% of those in AG single cohort. Among patients receiving combination therapy, the most common side effect was cytopenia (specifically leukopenia and anemia), generally present at grade 1–2 and typically developing within 3 months after treatment initiation. In comparison to the two monotherapy groups, the combination group exhibited a similar trend in the frequency of adverse effects. However, the HU plus AG combination therapy demonstrated a significantly lower incidence of grade 3–4 AEs compared to the other two (*p* = 0.008 for HU monotherapy vs. combination therapy and *p* < 0.01 for AG monotherapy vs. combination therapy). Six patients (five with grade 4 leukopenia/anemia and one with grade 2 dizziness) in the HU monotherapy group and three patients (two with grade 3 diarrhea, one with grade 3 peripheral edema, and one in grade 2 palpitation) in the AG monotherapy group were withdrawn from treatment due to adverse events, whereas no patients in the combination therapy group discontinued for this reason. There were no cases of drug-related death in any of the groups.

## 4. Discussion

In this study, we assessed the clinical outcomes of employing upfront combination therapy with HU and AG for intermediate or high-risk patients. To the best of our knowledge, this is the first study to assess the efficacy and safety of a low-dose combination regimen in cytoreduction therapy-naïve ET patients. Currently, there are no established guidelines for its utilization as a first-line approach. HU and AG function through distinct mechanisms of action and exhibit safety profiles that do not overlap with each other. Therefore, our findings suggest that the combination of HU and AG as initial therapy yielded comparable platelet count control and a relatively low incidence of severe drug-related toxicities, likely attributable to the reduction in daily dosages of both drugs compared to their individual administration. 

Some researchers reported that a combination regime of low-dose HU and AG achieved a 67% CR rate [11]. Christoforidou et al. reported a successful experience with combination therapy in 16 patients with ET and PV who were refractory or intolerant to monotherapy, with a 68.7% CR rate and a 31.3% PR rate [15]. In the EXELS study, a phase IV observational safety study involving high-risk ET patients undergoing cytoreductive therapy, approximately 80% of patients achieved a platelet count of 600 × 10^6^/L or less (≥PR according to ELN response criteria) six months after combination therapy [12]. In our study, patients receiving combination therapy showed similar overall response and CR rate to those previously published studies and were comparable to the rates observed in the monotherapy groups. Considering HU’s effectiveness in preventing arterial events and AG’s notable impact on reducing venous thrombosis, the combination of both drugs appears to be an effective strategy in preventing vascular complications, encompassing both arterial and venous thrombosis, which is the primary therapeutic goal for patients with ET. Based on the data from our study, the combination regimen in the frontline setting was able to achieve a clinical and laboratory response.

We also observed that patients in the combination therapy group and those treated with HU had a notably longer treatment duration compared to patients in the AG-treated group. This observation is consistent with the results from the EXELS trial. One potential explanation is that patients receiving AG were more swiftly transitioned to alternative regimens, possibly due to their failure to achieve a satisfactory platelet response within a relatively early timeframe, as illustrated in Figure 3, and/or due to a higher incidence of intolerable side effects. The median daily dose of AG (2 mg/day) in the AG monotherapy group in our study was relatively higher compared to another study (1.44 mg/day) where it was employed as a first-line therapy [17]. However, it is worth noting that in two randomized studies involving high-risk ET [5,6], AG demonstrated long-term platelet count control similar to that of HU. Nonetheless, it is generally poorly tolerated, especially at higher doses. In our study, no withdrawals from treatment occurred in the combination therapy group, while such events did occur in the monotherapy group. This highlights the impact of dose-dependent toxicities associated with higher doses of monotherapy, whether HU or AG, leading to suboptimal medication compliance and premature discontinuation of treatment. 

In relation to the safety data of this combination therapy, the observed drug-related toxicities in this study were generally in line with those reported in previously published studies [6,11,12,13,14,15]. Notably, in the ANAHYDRET trial, a phase III trial demonstrating non-inferiority of AG over HU in ET patients, cardiovascular side effects (specifically tachycardia and palpitation) were more frequently observed in the AG group, whereas cytopenia (leukopenia, anemia) was more frequently seen in the HU group [6]. Similarly, mucocutaneous pigmentation and cytopenia were more commonly observed in the HU group, while peripheral edema and gastrointestinal symptoms were more prevalent in the AG group. In the combination group, the incidence of overall toxicities was relatively lower than in each of the monotherapy groups in our present study and most of the drug-related adverse events were typically mild and manageable. This could be attributed to the low daily doses of each drug administered. Combining two drugs with distinct toxicities at low doses might lead to a decrease in the production of secondary metabolites associated with each drug, for example, the cytotoxic effect of free radicals on erythrocyte and granulocyte induced by HU [18], and the cardiovascular effect of phosphodiesterase inhibition induced by AG [19]. Consequently, this reduction may mitigate side effects such as cytopenia and cardiovascular events in patients. In our study, the daily doses of each drug in the combination group were significantly lower compared to those in the monotherapy groups (*p* = 0.01 for HU and *p* < 0.01 for AG), consistent with previous studies. For instance, Christoforidou et al. reported a successful experience of combination therapy in 16 patients with ET and PV who were refractory or intolerant to monotherapy, with median daily doses of HU and AG at 1285 mg and 1.5 mg, respectively [15]. Similarly, in the study by Ahn et al., the mean daily doses of HU and AG were 711 mg and 1.38 mg, respectively [11]. Furthermore, no treatment withdrawal due to drug-related toxicities was observed in the combination group compared to the monotherapy groups. These favorable safety profiles likely contributed to the significantly longer median duration of therapy observed in the combination group compared to the AG group. Given the low mean daily doses of combination HU and AG, it is plausible that a more favorable balance between toxicity (such as AG-related cardiovascular events and HU-induced cytopenia) and platelet count control could have been achieved. Hence, our results suggest that cytoreduction combination therapy with low-dose administration is a viable treatment option for ET patients in frontline settings, potentially circumventing side effects associated with high-dose treatments.

Similar to our study, there is a paucity of clinical parameters that can predict the treatment response of HU or AG and prognosis in ET. Currently, pharmacogenomics can play an important role in identifying responders and non-responders to treatment [20]. The expression of the ribonucleotide reductase M1 (RRM1) gene may be associated with the efficacy of HU treatment in some cancers, and some researchers have suggested that overexpression of the RRM1 gene might confer resistance to HU [21]. By inhibiting the phosphodiesterase 3A (PDE3A) gene, AG can regulate cAMP concentrations and suppress platelet production [22]. While still in the research phase, there is the potential utility of targetable pharmacogenomic biomarkers, such as the RRM1 gene for HU, and the PDE3A gene for AG, in predicting clinical responses.

This study has a potential limitation due to its retrospective study design. The small sample size of patients receiving the combination therapy of HU and AG introduced an inherent selection bias that could not be entirely avoided. However, any patients with incomplete data, encompassing parameters such as complete blood counts, adverse events, and survival, were excluded from the analysis. Furthermore, the median follow-up of 3.1 years for ET patients in our study may not be entirely sufficient to comprehensively assess the long-term effectiveness and diverse complications associated with combination therapy, including thromboembolic events, infections, and secondary malignancies. While data on JAK2 mutation status were universally available, only a small subset of patients had data regarding the status of myeloproliferative leukemia virus oncogene (MPL) and calreticulin (CARL) mutations, which are estimated to occur in approximately 4% and 20% of ET patients. Going forward, we are planning to conduct a prospective study aiming to investigate the long-term outcomes of this combination therapy and identifying prognostic factors, including the status of all three mutations.

## 5. Conclusions

Our findings indicated that HU plus AG combination therapy, when administered as a first-line treatment, demonstrated comparable clinical and laboratory responses with a low incidence of severe side effects in comparison to conventional monotherapy. This study suggests that such combination therapy may constitute a viable and effective treatment approach in the frontline setting for patients with intermediate or high risk. These results offer valuable insights for guiding future prospective investigations. Additionally, further research is warranted to discern predictive markers of treatment response, such as pharmacogenomic biomarkers, aiding in the identification of specific ET patients who are likely to derive substantial benefits from upfront HU and AG combination therapy.

## Figures and Tables

**Table 1:** Baseline characteristics at the time of treatment initiation.

Parameters	^1^ HU + ^2^ AG (*n* = 21)	HU Alone (*n* = 151)	AG Alone (*n* = 69)	*p*-Value
Age at diagnosis, median (range)	62 (26–87)	64 (29–71)	59 (31–82)	0.068
Male, *n* (%)	9 (42.9)	70 (46.4)	29 (42)	0.485
Female, *n* (%)	12 (57.1)	81 (53.6)	40 (58)	
^3^ CBC profiles at diagnosis				0.351
Hemoglobin, median (range), g/dL	14.6 (8.3–18.5)	13.5 (8.6–19.2)	13.4 (9.1–18.2)	
^4^ WBC, median (range), ×109/L	8.7 (4.5–21)	9.2 (5.1–23)	7.8 (4.8–19.5)	
Platelet, median, (range), ×109/L	912 (520–1720)	970 (620–1650)	920 (540–1890)	
High ^5^ LDH level, *n* (%)	9 (42.9)	64 (42.4)	31 (44.9)	0.236
Hepatosplenomegaly, *n* (%)	6 (28.5)	39 (25.8)	15 (21.7)	0.478
^6^ IPSET-thrombosis risk, *n* (%)				0.081
Intermediate risk	9 (42.9)	84 (55.6)	46 (66.7)	
High risk	12 (57.1)	67 (44.4)	23 (33.3)	
Microvascular symptoms, *n* (%)	6 (28.6)	41 (27.2)	24 (34.8)	0.325
*JAK2 V617F* mutation, *n* (%)	14 (66.7)	85 (56.3)	36 (52.2)	0.489
History of thrombotic events, n (%)				0.411
Arterial event	2 (9.5)	20 (13.2)	8 (11.6)	
Venous event	2 (9.5)	11 (7.3)	4 (5.8)	
History of any bleeding events, *n* (%)	1 (4.8)	2 (1.3)	1 (1.4)	0.588
Presence of cardiovascular risk, *n* (%)	15 (71.4)	78 (51.7)	41 (59.4)	0.314
Hypertension	11 (52.4)	59 (41.3)	35 (50.8)	
Diabetes mellitus	8 (38.1)	42 (27.9)	24 (34.8)	
Dyslipidemia	9 (42.9)	36 (25.2)	10 (14)	
Anti-platelet medication, *n* (%)	11 (52.4)	52 (34.4)	19 (27.5)	0.157

^1^ HU, hydroxyurea; ^2^ AG, anagrelide; ^3^ CBC, complete blood cell count; ^4^ WBC, white blood cell; ^5^ LDH, lactate dehydrogenase; ^6^ IPSET-thrombosis, International Prognostic Score of thrombosis in World Health Organization-essential thrombocythemia.

**Table 2:** Summary of treatment and outcome in each treatment group.

	^1^ HU + ^2^ AG (*n* = 21)	HU Alone (*n* = 151)	AG Alone (*n* = 69)	*p*-Value
Daily dose, mg/day				
HU, mean (range)	1142 (500–2000)	1653 (500–3000)		0.01
AG, mean (range)	1.45 (1.0–2.5)		1.82 (1.0–3.0)	<0.01
Duration of therapy, day				0.022
Median (range)	608 (258–1584)	674 (265–1757)	539 (198–1678)	
Best overall response, *n* (%)				0.468
Complete response	12 (57.1)	97 (64.1)	41 (59.4)	
Partial response	8 (38.1)	50 (33.1)	24 (34.8)	
No response	1 (4.8)	4 (2.6)	4 (5.8)	
Regimen switch during treatment				0.085
HU alone	2 (9.5)	-	5 (7.2)	
AG alone	3 (14.3)	11 (7.3)	-	
HU plus AG combination	-	16 (10.6)	19 (27.5)	
Treatment discontinuation	0 (0)	8 (5.2)	3 (4.3)	
Reason for regimen switch				
Lack of efficacy	1 (4.8)	23 (15.2)	21 (30.4)	<0.01
Adverse events	2 (9.5)	7 (4.6)	3 (4.3)	0.098
Transformation	0 (0)	4 (2.8)	2 (2.9)	0.365
Physician’s choice	2 (9.5)	0 (0)	0 (0)	0.458
Thrombosis events after treatment				0.088
Arterial events, *n* (%)	1 (4.8)	3 (2)	2 (2.9)	
^3^ CVA/^4^ MI	1 (4.8)/0 (0)	1 (0.7)/1 (0.7)	1 (1.4)/0 (0)	
Others	0 (0)	1 (0.7)	1 (1.4)	
Venous events, *n* (%)	0 (0)	6 (4.0)	1 (1.4)	
^5^ DVT/^6^ PTE	0 (0)	3 (2)/2 (1.4)	0 (0)/1 (1.4)	
Others	0 (0)	1 (0.7)	0 (0)	
Bleeding events after treatment, *n* (%)	0 (0)	1 (0.7)	2 (2.9)	0.369
Transformation, *n* (%)	1 (4.8)	7 (4.6)	2 (2.9)	0.536
Myelofibrosis	1 (4.8)	5 (3.3)	2 (2.9)	
Acute myeloid leukemia	0 (0)	1 (0.7)	0 (0)	
Myelodysplastic syndrome	0 (0)	1 (0.7)	0 (0)	
Non-hematologic malignancy, *n* (%)	1 (4.8)	1 (0.7)	0 (0)	0.568

^1^ HU, hydroxyurea; ^2^ AG, anagrelide; ^3^ CVA, cerebrovascular accident; ^4^ MI, myocardial infarction; ^5^ DVT, deep vein thrombosis; ^6^ PTE, pulmonary thromboembolism.

**Table 3:** Summary of adverse events.

Adverse Events	^1^ HU + ^2^ AG (*n* = 21)		HU Alone (*n* = 151)		AG Alone (*n* = 69)	
	Any Grade	Grade 3–4	Any Grade	Grade 3–4	Any Grade	Grade 3–4
Mucocutaneous manifestation	6 (28.6%)	0	65 (43.0%)	0	0	0
Leukopenia	4 (19.0%)	0	89 (58.9%)	16 (10.6%)	2 (2.9%)	1 (1.4%)
Anemia	3 (14.3%)	0	92 (60.9%)	10 (6.6%)	0	0
Diarrhea	1 (4.8%)	0	6 (4.0%)	3 (2.0%)	6 (8.7%)	2 (2.9%)
Abdominal discomfort/dyspepsia	2 (9.5%)	0	26 (17.2%)	3 (2.0%)	8 (11.6%)	3 (4.3%)
Febrile sensation	1 (4.8%)	0	3 (2.0%)	0	0	0
Peripheral edema	2 (9.5%)	0	1 (0.7%)	0	12 (17.4%)	2 (2.9%)
Dizziness	2 (9.5%)	0	11 (7.3%)	0	3 (4.3%)	1 (1.4%)
Headache	1 (4.8%)	0	9 (6.0%)	0	7 (10.1%)	3 (4.3%)
Palpitation	2 (9.5%)	0	0	0	16 (23.2%)	7 (10.1%)
Chest pain/discomfort	2 (9.5%)	0	3 (2.0%)	1 (0.7%)	19 (27.5%)	5 (7.2%)
Leg ulcer	0	0	1 (0.7%)	0	0	0
Fatigue	9 (42.9%)	0	38 (25.2%)	2 (1.3%)	29 (42.0%)	3 (4.3%)
Others	4 (19.0%)	0	14 (9.3%)	0	6 (8.7%)	0
Total events	39	0	358	35	108	27

^1^ HU, hydroxyurea; ^2^ AG, anagrelide.

## Data Availability

Datasets used and analyzed in this study are available from the corresponding author on reasonable request.
